# A Comparative Assessment of Intraocular Pressure Changes After Aflibercept 8 mg and Faricimab-svoa Intravitreal Injections in Wet Age-Related Macular Degeneration

**DOI:** 10.7759/cureus.88617

**Published:** 2025-07-23

**Authors:** Panos Gartaganis, Norhan Khamis, Aseel Hamoud Bedan, Zahira Driouich, Muhammad Omar Ashraf, Sirad Abucar Osman, Saad Younis

**Affiliations:** 1 Medical Retina Service, Western Eye Hospital, Imperial College Healthcare NHS Trust, London, GBR; 2 Nursing, Western Eye Hospital, Imperial College Healthcare NHS Trust, London, GBR

**Keywords:** aflibercept 8mg intravitreal injection, anti-vegf treatment, faricimab intravitreal injection, intraocular pressure (iop), wet-aged related macular degeneration

## Abstract

Introduction

A standard practice for addressing wet age-related macular degeneration (WetAMD) is to deliver anti-vascular endothelial growth factor (anti-VEGF) intravitreal injections. Formulations with higher concentrations, such as aflibercept 8 mg (Eylea HD; Bayer AG, Leverkusen, Germany), which are administered in larger volumes, may raise concerns about potential increases in intraocular pressure (IOP) and other ocular complications. The objective of this study was to evaluate and compare short-term IOP changes following intravitreal injection of aflibercept 8 mg (Eylea HD, 0.07 mL) versus faricimab-svoa (Vabysmo, 0.05 mL; Roche Pharma, Basel, Switzerland/Genentech, South San Francisco, CA, USA) in patients with WetAMD.

Methods

A retrospective observational analysis was conducted involving patients with WetAMD who received intravitreal injections of aflibercept 8 mg (n = 64 eyes) or faricimab-svoa (n = 73 eyes). IOP was measured before the injection and 30 minutes after. The research recorded lens condition, the need for paracentesis, and the application of Iopidine drops. Patients were categorized into phakic and pseudophakic subgroups, and further stratified based on post-injection IOP levels: <20 mmHg, 20-25 mmHg, 25-30 mmHg, and >30 mmHg.

Results

Both treatment groups showed notable increases in IOP at 30 minutes after injection (Eylea HD: +4.50 ± 4.32 mmHg, Vabysmo: +3.66 ± 5.20 mmHg; p = 0.083). However, there were no instances requiring paracentesis, and only one patient from each group needed Iopidine drops. Pseudophakic patients experienced slightly higher IOP increases (Eylea HD: +4.71 ± 4.18 mmHg, Vabysmo: +4.55 ± 5.65 mmHg; p = 0.784) compared to phakic patients. The majority of patients maintained IOP levels under 30 mmHg. Gender distribution was 49% male and 51% female.

Conclusions

Intravitreal injections of aflibercept 8 mg and faricimab-svoa caused a small and temporary increase in IOP and there were no cases requiring urgent management. Our results confirm the short-term ocular safety of aflibercept 8 mg and faricimab-svoa for the treatment of WetAMD and highlight the need for individualized monitoring for patients at risk of increased IOP.

## Introduction

Age-related macular degeneration (AMD) has emerged as a leading cause of visual impairment in older adults, necessitating innovative treatment approaches to manage its progression effectively. The arrival of anti-vascular endothelial growth factor (anti-VEGF) therapies, such as aflibercept and ranibizumab, has marked a significant advancement in the therapeutic landscape for AMD, diabetic macular oedema (DMO), and retinal vein occlusion (RVO) [1-3]. These agents target the overexpression of VEGF - a crucial factor in neovascularization - thereby stabilizing and, in some cases, improving visual acuity in affected patients.

However, while the efficacy of these treatments has been well documented, concerns regarding their safety profile - particularly related to intraocular pressure (IOP) elevation following intravitreal injections - continue to garner attention [4,5]. Recent studies have indicated that IOP spikes can occur shortly after administration of intravitreal anti-VEGF agents, raising questions about the implications for patients with pre-existing glaucoma or ocular hypertension [6,7].

Understanding the multifactorial determinants of IOP changes post-injection is critical for optimizing patient outcomes, especially given the recent advancements in formulations and delivery systems of these therapies. For example, prefilled syringes have been shown to demonstrate variability in the volumes of fluid delivered, which could affect IOP responses [8,9]. Additionally, studies evaluating the long-term effects of repeated injections on IOP have underscored the importance of continuous monitoring and management strategies to mitigate risks associated with elevated pressures [10,11].

Considering these findings, the current study aims to measure and compare the short-term IOP changes associated with intravitreal injection of aflibercept 8 mg versus faricimab-svoa in patients with wet age-related macular degeneration (WetAMD), and to illustrate the need for IOP monitoring and the advantages of individualized treatment options for patients at higher risk. Additionally, it takes into account new data regarding the safety and efficacy of recently introduced treatment options - specifically faricimab-svoa and aflibercept 8 mg - in clinical practice [12-14]. Secondary objectives of this study included evaluating the relationship between lens status (phakic vs. pseudophakic) and IOP response, as well as evaluating any additional management required (e.g., Iopidine administration or paracentesis after intravitreal injection).

## Materials and methods

Study design

This comparative retrospective observational study examined data from patients with WetAMD who received treatment with aflibercept 8 mg or faricimab-svoa at our facility between April and June 2024.

Patient selection

Inclusion Criteria

Participants were eligible for inclusion if they were diagnosed with WetAMD, received intravitreal injections of either aflibercept 8 mg or faricimab-svoa between April and June 2024, had complete pre- and post-injection IOP data, had no prior ocular surgery other than cataract extraction, and could provide informed consent for data analysis.

Exclusion Criteria

Participants were excluded if they had a history of glaucoma or ocular hypertension, had undergone other intraocular surgeries such as vitrectomy or trabeculectomy, had incomplete clinical records or missing IOP measurements, or had concurrent ocular conditions such as uveitis or corneal pathology that could interfere with IOP measurement.

Study procedures and data source 

This study was a retrospective observational study and used data primarily from the electronic medical records of the Western Eye Hospital. All clinical procedures were performed as part of routine care, using aflibercept 8 mg or faricimab-svoa, in accordance with institutional protocol. Although routine measurements of IOP were standard clinical assessment pre-injection, we did not actively encourage or request baseline IOP measurements for this study. However, we did prospectively and uniformly measure IOP at 30 minutes post-injection for an internal departmental audit, which addressed the short-term ocular safety of aflibercept 8 mg using a real-world practice assessment.

No experimental interventions were applied, and all information was collected retrospectively, including lens status, demographic information, and any post-injection interventions such as Iopidine (apraclonidine) or paracentesis. Assessment of IOP was conducted by multiple trained healthcare professionals (nurses), with no blinding of clinicians (observational bias must be considered). We did not formally evaluate power or perform a sample size calculation, as we included all eligible cases within a specified time frame. No patients were contacted or informed of any procedures beyond routine care, and all data extraction was conducted in accordance with ethical principles for retrospective research.

Intravitreal injection technique

Injections were performed under sterile conditions, in accordance with the established clinical protocol at our institution. The eyelids and eye surfaces were cleaned with 5% povidone-iodine, and a sterile drape, along with a lid speculum, was used for each operation. Aflibercept 8 mg (Eylea HD; Bayer AG, Leverkusen, Germany) was prepared by drawing the entire contents of the vial (30.1 mg/0.263 mL) into a 1 mL sterile syringe fitted with an 18-gauge needle. A 30-gauge needle was then attached, inserted up to the hub, and the excess medication was expelled, resulting in a final volume of 0.07 mL (8 mg) to be administered into the vitreous. In a similar way, injections of faricimab-svoa (Vabysmo; Roche Pharma, Basel, Switzerland/Genentech, South San Francisco, CA, USA) were prepared using the same technique. Specifically, 28.8 mg/0.24 mL was withdrawn from the vial, and then a volume of 0.05 mL (6 mg) was administered into the vitreous.

IOP measurement and analysis

IOP was measured using the iCare IC200 Tonometer (iCare Finland Oy, Helsinki, Finland), with readings collected pre-injection and 30 minutes post-injection. Additional data included lens status (categorized as phakic or pseudophakic), necessity for therapeutic interventions (such as paracentesis or administration of Iopidine drops), and demographic characteristics. IOP values post-injection were stratified into categories: <20 mmHg, 20-25 mmHg, 25-30 mmHg, and >30 mmHg. Statistical analyses were carried out using IBM SPSS Statistics for Windows, Version 28.0 (released 2021; IBM Corp., Armonk, NY, USA). Descriptive statistics were used to summarize the demographic and clinical characteristics, including means and standard deviations for continuous variables. Group comparisons of IOP changes were tested using independent-samples t-tests. The Shapiro-Wilk test was used to check for normality of data distribution prior to each parametric test. A p-value of <0.05 was used as the cutoff for statistical significance. Additionally, 95% confidence intervals (CIs) were provided for the mean IOP changes in each subgroup to estimate the precision of the observed effects.

Limitations

This study was designed as a retrospective analysis but was complemented by a prospectively planned departmental audit of post-injection IOP measurements. Therefore, the study did not conduct formal sample or power calculations prior to data collection. IOP measurements were performed by trained healthcare professionals (nurses) using the iCare IC200 Tonometer and were conducted in a non-blinded manner, which could contribute to observer bias. The data collected were pooled from a single tertiary care centre over a potential three-month period, which could restrict external validity. Patients with incomplete IOP records or unclear lens status were excluded from analysis; however, there was no imputation of any missing data.

Ethics statement

This study was executed in line with the principles outlined in the Declaration of Helsinki. Patient confidentiality was maintained, and all data were anonymized prior to analysis. According to the hospital audit committee’s guidelines of our trust, there is no requirement for an IRB decision letter when a clinical audit is deemed acceptable for implementation.

## Results

**Figure 1:**
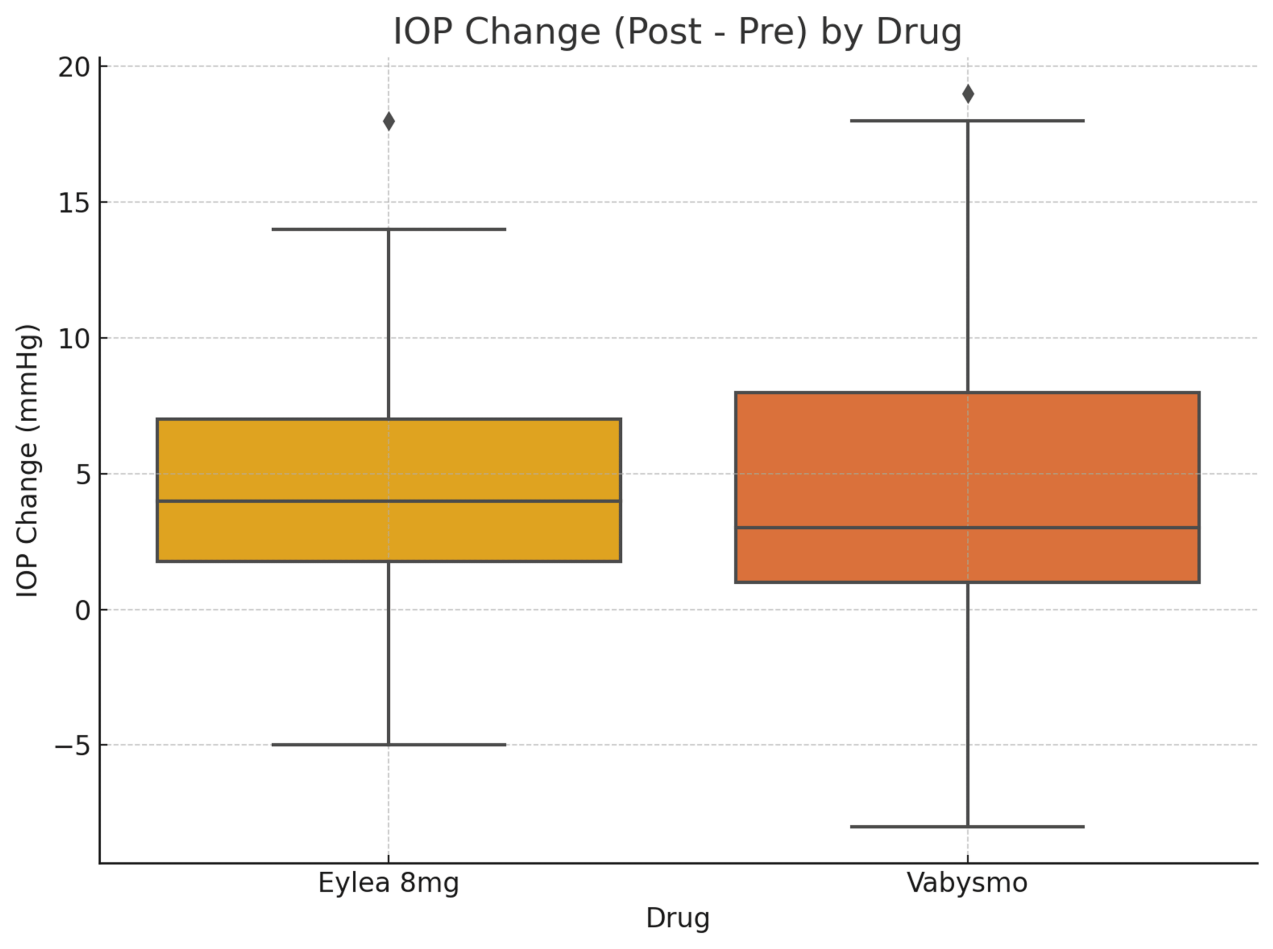
IOP changes by drug Boxplot of IOP changes by drug. This visualizes the distribution and range of IOP change from baseline to 30 minutes post-injection for Eylea 8 mg vs. Vabysmo. The error bars in this boxplot are the whiskers, which typically represent the range within 1.5 × IQR (interquartile range) from the first (Q1) and third (Q3) quartiles. Outliers beyond this range are shown as individual points. The box itself spans Q1 to Q3, with a horizontal line at the median. IOP, intraocular pressure

**Figure 2:**
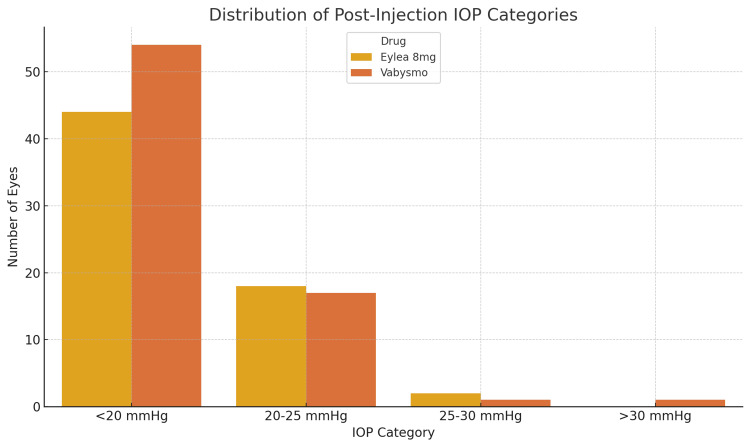
Distribution of patients by post-injection IOP category for each drug Countplot showing the distribution of patients by IOP category (<20, 20-25, 25-30, >30 mmHg), post-injection, for each drug. IOP, intraocular pressure

A total of 137 eyes were assessed, with 64 treated with aflibercept 8 mg and 73 treated with faricimab-svoa. The gender distribution consisted of 48% male and 52% female. The mean pre-injection IOP was similar for both groups (Eylea HD: 12.13 ± 3.27 mmHg, Vabysmo: 12.56 ± 3.20 mmHg; p = 0.442). A significant but moderate increase in IOP was observed during the post-injection assessment after 30 minutes (Eylea HD: +4.50 ± 4.32 mmHg, Vabysmo: +3.66 ± 5.20 mmHg; p = 0.083). This IOP change approaches statistical significance, although it does not reach the conventional threshold (p < 0.05). This suggests a trend toward a difference in IOP change between the two groups, but it is not strong enough to indicate a statistically significant difference based on the available data (Figure 1). Furthermore, the term "moderate increase" describes an elevation in IOP that would not, under normal circumstances, exceed the threshold for intervention (>30 mmHg). Notably, most patients maintained IOP levels below 25 mmHg, underscoring the treatment's short-term safety profile (Figure 2).

**Figure 3:**
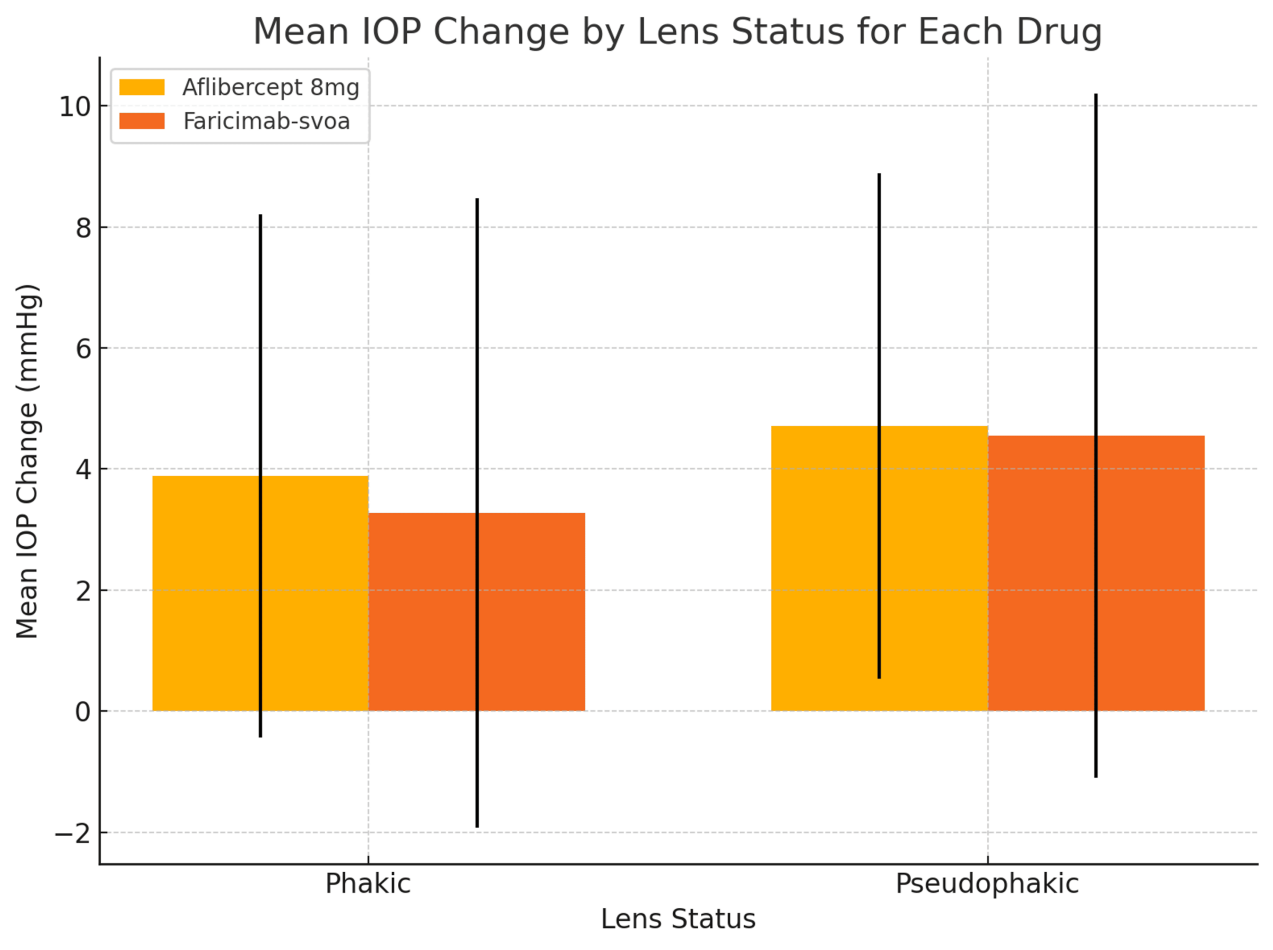
Mean IOP change by lens status across drug groups Bar plot representing mean IOP change based on lens status (phakic vs. pseudophakic) across both drug groups. The vertical error bars in this bar plot represent the standard deviation (SD) of the IOP change within each group, defined by lens status and drug. IOP, intraocular pressure

Analysis of lens status showed that pseudophakic eyes in both treatment groups exhibited slightly higher IOP increases than phakic eyes. This can be explained by the altered anterior chamber dynamics and changes in ocular rigidity that can occur following cataract surgery. Specifically, for aflibercept 8 mg, pseudophakic eyes experienced an increase of +4.71 mmHg, compared to +3.89 mmHg in phakic eyes (p = 0.784). A similar pattern was found for faricimab-svoa, with pseudophakic eyes showing an increase of +4.54 mmHg versus +3.28 mmHg for phakic patients (p = 0.325). The IOP change differences between phakic and pseudophakic eyes were not statistically significant in either treatment group. The pseudophakic eyes did, however, have higher increases in IOP, but this was considered too variable to be regarded as a statistically meaningful difference (Figure 3).

The average IOP changes 30 minutes post-injection were 4.50 mmHg (95% CI: 3.42 to 5.58) for the aflibercept 8 mg group, and 3.66 mmHg (95% CI: 2.45 to 4.87) for the faricimab-svoa group. When stratified by lens status, phakic eyes in the aflibercept group were noted to have a mean IOP increase of 3.89 mmHg (95% CI: 2.33 to 5.45), and pseudophakic eyes had a mean increase of 4.70 mmHg (95% CI: 3.19 to 6.21). There was a similar trend in the faricimab group, with phakic eyes noticing a mean change of 3.28 mmHg (95% CI: 1.52 to 5.04), and pseudophakic eyes a mean change of 4.54 mmHg (95% CI: 2.66 to 6.42). These CIs support that a mild elevation of IOP is reflected across both treatment groups and lens status subgroups, with no statistically significant differences between lens statuses.

The necessity for additional therapeutic interventions remained minimal throughout the study. No cases required paracentesis, illustrating that significant IOP elevations necessitating invasive measures were absent. Moreover, only one patient in each group required Iopidine (apraclonidine) drops, indicating that the transient IOP increases post-injection did not lead to complications warranting further treatment.

## Discussion

This study highlights the significance of recognizing fluctuations in IOP following intravitreal anti-VEGF injections. While these treatments are crucial for managing retinal diseases like AMD and DMO, transient and potentially sustained IOP elevation is a concern - especially for patients with glaucoma or ocular hypertension. Our findings reveal variability in patient responses to different anti-VEGF agents due to factors such as formulation differences, injection techniques, and individual anatomical considerations. Clinicians ought to employ a personalized strategy by performing comprehensive evaluations before treatment and customized monitoring after injections, while also informing patients about the signs of increased IOP. The introduction of new treatments, like faricimab-svoa and aflibercept 8 mg, presents both opportunities and challenges regarding their effects on IOP. Future research needs to explore the mechanisms behind IOP changes post-injection and identify effective mitigative strategies, considering factors such as ocular rigidity and pre-existing conditions. 

The results of this study add to the expanding research on the intricate connection between intravitreal anti-VEGF therapies and variations in IOP. While several studies have documented transient IOP spikes post-injection, our results emphasize the variability in individual patient responses, highlighting the need for personalized treatment plans. The variations seen in IOP after administration of different agents indicate that both pharmacological features and injection methods can have a significant impact on clinical outcomes [15,16]. One significant concern is the risk of prolonged elevation of IOP, which can adversely affect patients who already have ocular hypertension or glaucoma [5,6]. The implications of these findings extend beyond immediate IOP spikes; they necessitate a re-evaluation of post-injection protocols and risk stratification for individuals receiving frequent intravitreal treatments. In addition, as new anti-VEGF medications become available, with extended dosing schedules and enhanced effectiveness, it will be important to comprehend their impact on IOP for the purpose of refining treatment plans [12,17]. Also, continuous research will be crucial in clarifying the mechanisms that lead to changes in IOP after intravitreal injections. Factors such as ocular rigidity, anterior chamber depth, and injection technique may intertwine to significantly influence patient outcomes [18,19]. Additionally, the role of consistent monitoring and patient education on IOP management strategies will be pivotal in ensuring that the benefits of anti-VEGF therapies outweigh the risks associated with potential ocular hypertension [10,20].

In our clinic, we assess IOP and visual acuity pre-treatment - or pre-injection - for every patient, whether they receive an injection or not. This baseline assessment helps identify patients who may be at greater risk for significant increases in IOP during treatment. For patients with a history of glaucoma or ocular hypertension who are actively undergoing treatment, we have started proactively administering a drop of Iopidine (apraclonidine) both before and after injection, which we feel helps mitigate potentially concerning IOP spikes. Likewise, for patients at a known or suspected risk of IOP elevation, or with a concomitant history that predisposes them to IOP increases, we routinely measure IOP approximately 20 minutes after injection to safely and effectively capture any immediate post-injection changes. 

We do note that the strength of our conclusions is limited by the limitations of our study. The hybrid design (retrospective chart review with prospectively planned IOP measurements) inevitably creates variation in data consistency, due to incomplete or biased data. Also, even though a 30-minute post-injection interval captured the initial or acute changes in IOP, we cannot comment on delayed or cumulative increases in IOP that are clinically relevant in patients receiving long-term anti-VEGF therapy. These limitations are amplified by our study design, including the absence of power calculations, poor generalizability due to single-centre data, and lack of blinding of IOP measurements. Since this study was conducted at a single institution and data were collected over a short time period, important external validity is lost. Lastly, this study was neither designed nor powered to evaluate rare adverse events or longer-term safety outcomes; therefore, our findings should be interpreted with caution.

The importance of sustained collaboration with ophthalmologists, clinical researchers, and advocates for patient safety in further developing treatment restrictions to protect, whenever possible, ocular health - without preventing therapeutic advantage to patients - cannot be overstated. For example, any elevations in IOP post anti-VEGF therapy should still be assessed after injection, especially for patients at high risk (e.g., those with glaucoma, ocular hypertension, or altered anterior segment anatomy). The data reported in this manuscript show mild and transient elevations in IOP, without the need for any rescue intervention, thereby establishing the short-term safety of aflibercept 8 mg and faricimab-svoa. However, ongoing clinical diligence, individual monitoring and vigilance, and future research related to cumulative IOP are both plausible and essential for advancing evidence-based care and maximizing retinal disease outcomes.

## Conclusions

Overall, this study has demonstrated that there is a slight, transient elevation in IOP measured 30 minutes after the injection, following the use of both aflibercept 8 mg and faricimab-svoa, which do not produce clinically significant pathological spikes that warrant any form of intervention. Such data are consistent with the short-term safety profile of these agents in the therapeutic management of WetAMD, as well as with findings captured in real-world clinical practice. Clarity will be maintained for clinicians to exercise vigilance - particularly in those with known or suspected risk factors for IOP elevation - and through thorough follow-up engagements. Future studies into the long-term effects of repeated injections on IOP, and the application of preventive measures such as prophylactic IOP-lowering medications and modified injection techniques, would be valuable. Wherever possible, the evidence base that will ultimately inform clinicians' decisions in retinal disease therapy should be strengthened with multicentre, prospective, and blinded studies.
